# Matched comparison between external aortic root support and valve-sparing root replacement

**DOI:** 10.1136/heartjnl-2022-321840

**Published:** 2023-01-17

**Authors:** Lucas Van Hoof, Marie Lamberigts, Dries Noé, Ismail El-Hamamsy, Emmanuel Lansac, Jolanda Kluin, Laurent de Kerchove, John Pepper, Tom Treasure, Bart Meuris, Filip Rega, Peter Verbrugghe

**Affiliations:** 1 Cardiac Surgery, KU Leuven University Hospitals Leuven, Leuven, Belgium; 2 Cardiovascular Surgery, Mount Sinai Hospital, New York, New York, USA; 3 Cardiothoracic Surgery, University Hospital Pitié Salpêtrière, Paris, France; 4 Cardiothoracic Surgery, Amsterdam UMC Locatie AMC, Amsterdam, The Netherlands; 5 Cardiovascular Surgery, Cliniques Universitaires Saint-Luc, Brussels, Belgium; 6 Cardiac Surgery, Royal Brompton and Harefield NHS Trust, London, UK; 7 Cardiovascular Biomedical Research Unit (BRU), NIHR Imperial Biomedical Research Centre, London, UK; 8 Clinical Operational Research Unit, University College London, London, UK; 9 AVIATOR, Heart Valve Society, Beverly, Massachusetts, USA

**Keywords:** Aortic Aneurysm, Marfan Syndrome, Aortic Valve Insufficiency

## Abstract

**Objectives:**

Differences in indication and technique make a randomised comparison between valve-sparing root replacement (VSRR) and personalised external aortic root support (PEARS) challenging. We performed a propensity score (PS)-matched comparison of PEARS and VSRR for syndromic root aneurysm.

**Methods:**

Patients in the PEARS 200 Database and Aortic Valve Insufficiency and ascending aorta Aneurysm InternATiOnal Registry (undergoing VSRR) with connective tissue disease operated electively for root aneurysm <60 mm with aortic regurgitation (AR) <1/4 were included. Using a PS analysis, 80 patients in each cohort were matched. Survival, freedom from reintervention and from AR ≥2/4 were estimated using a Kaplan-Meier analysis.

**Results:**

Median follow-up was 25 and 55 months for 159 PEARS and 142 VSRR patients. Seven (4.4%) patients undergoing PEARS required an intervention for coronary injury or impingement, resulting in one death (0.6%). After VSRR, there were no early deaths, 10 (7%) reinterventions for bleeding and 1 coronary intervention. Survival for matched cohorts at 5 years was similar (PEARS 98% vs VSRR 99%, p=0.99). There was no difference in freedom from valve or ascending aortic/arch reintervention between matched groups. Freedom from AR ≥2/4 at 5 years in the matched cohorts was 97% for PEARS vs 92% for VSRR (p=0.55). There were no type A dissections.

**Conclusions:**

VSRR and PEARS offer favourable mid-term survival, freedom from reintervention and preservation of valve function. Both treatments deserve their place in the surgical repertoire, depending on a patient’s disease stage. This study is limited by its retrospective nature and different follow-ups in both cohorts.

**WHAT IS ALREADY KNOWN ON THIS TOPIC**

- While valve-sparing root replacement (VSRR) is the established treatment for syndromic root aneurysm, there remains a cumulative risk of aortic valve reintervention. In personalised external aortic root support (PEARS), the dilated aorta is supported using a bespoke mesh, optimally respecting valve anatomy. To date, no type A dissections have been observed after PEARS.

**WHAT THIS STUDY ADDS**

- VSRR and PEARS both seem to offer favourable mid-term survival, freedom from reintervention and preservation of valve function in syndromic root aneurysm with near-normal valve function. Our study indicates that an earlier intervention in the disease progression via PEARS may be justified with a low probability of developing aortic events if the necessary attention goes to the coronary anatomy.

**HOW THIS STUDY MIGHT AFFECT RESEARCH, PRACTICE OR POLICY**

- Long-term data are needed to determine the role of all possible treatments in the surgical repertoire. Improved prediction of aortic complications will further help guide patient selection. A shared decision-making process should involve weighing the risk of watchful waiting against the potential risks of all available surgical treatments.

VSRR and PEARS both seem to offer favourable mid-term survival, freedom from reintervention and preservation of valve function in syndromic root aneurysm with near-normal valve function. Our study indicates that an earlier intervention in the disease progression via PEARS may be justified with a low probability of developing aortic events if the necessary attention goes to the coronary anatomy.

## Introduction

**Figure 1:**
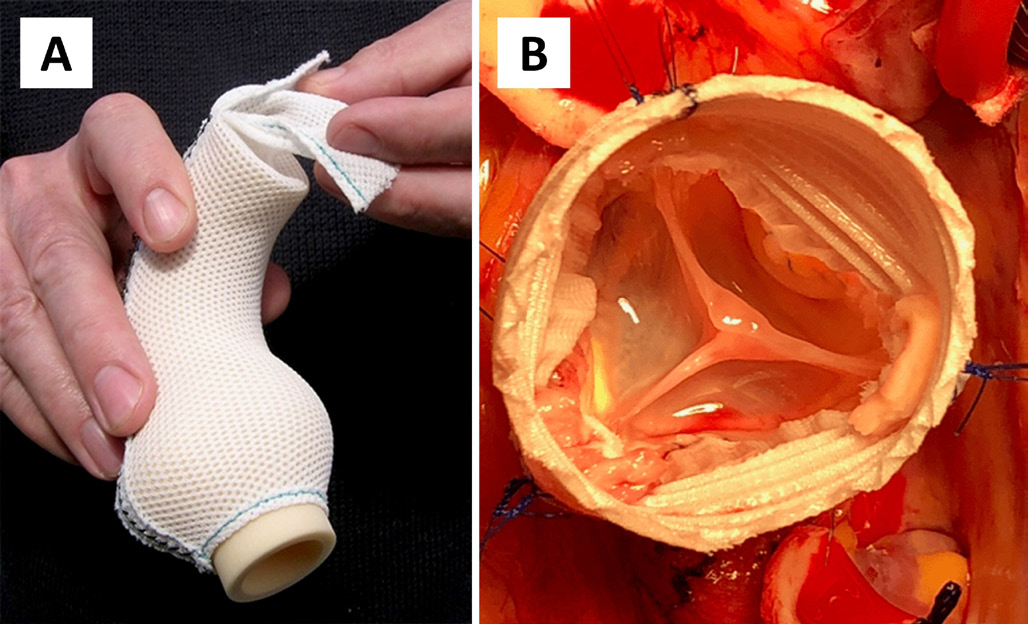
(A) ExoVasc implant used to stabilise the ascending aorta during personalised external aortic root support surgery. (B) Intraoperative photograph of valve-sparing root replacement. The corrugations of the relatively rigid structure of the low-porosity vascular graft can be seen. Illustration provided by Exstent.

Valve-sparing root replacement (VSRR) is an established surgical treatment for aortic root aneurysm.^1^ Depending on patient selection and surgeon experience, excellent freedom from reintervention with a low incidence of valve-related events can be achieved in Marfan syndrome (MFS) and other connective tissue diseases (CTDs).^2^ There remains, however, a cumulative risk of reintervention on the aortic valve and distal aorta.^3 4^ Personalised external aortic root support (PEARS) is a total tissue-preserving alternative to VSRR which involves the use of a bespoke mesh, the ExoVasc implant (figure 1), to stabilise the aorta from the ventriculoaortic junction to the origin of the brachiocephalic trunk.^5 6^ This emerging procedure has been applied primarily in patients with syndromic root aneurysm between 40 and 50 mm and at most mild (grade 1/4) aortic regurgitation (AR) as previously reported in *Heart* in 2014 and 2021.^6 7^ Because the dilated aorta is left in place, patients undergoing PEARS are typically operated in an earlier disease stage than if they would undergo VSRR. No dissections have been observed in the supported segment of aorta yet continued follow-up is needed.^7^ Differences in indication, timing and surgical technique make it difficult to define a group of patients where there is equipoise for a randomised comparison between PEARS and VSRR.^8^ PEARS is typically a pre-emptive procedure performed in the preclinical phase, while VSRR is an established prophylactic operation performed when a diameter threshold is met.^1^ We aimed to compare demographics and outcomes of patients undergoing PEARS and VSRR for syndromic root aneurysm with at most mild AR and to perform a propensity score (PS)-matched analysis to discover the magnitude of difference for available outcome measures.

## Methods

### Study design

We present a multicentre study using two existing databases: the ‘PEARS 200’ and ‘AVIATOR’ Database (Aortic Valve Insufficiency and ascending aorta Aneurysm InternATiOnal Registry), both containing prospectively and retrospectively collected data.^7 9^ The AVIATOR Project was initiated by the Heart Valve Society as an international registry collecting data on patients undergoing surgery on the proximal aorta, with patients consenting.^9^ A proposal to the AVIATOR scientific committee request permission for a subgroup analysis (RP#13) was approved. Perioperative outcomes on all patients undergoing PEARS are collected prospectively by Exstent. In the context of an earlier study, we retrospectively collected clinical follow-up data for the first 200 consecutive PEARS operations for root aneurysm worldwide.^7^

### Data collection and study endpoints

From both databases, patients with CTD (MFS, Loeys-Dietz syndrome and ACTA2 mutations) undergoing elective surgery for aortic root aneurysm <60 mm diameter with at most mild AR (grade 0/4 or 1/4) were extracted. Patients with ascending aortic dissection or endocarditis were excluded. The primary endpoint was mid-term survival. Secondary endpoints were in-hospital mortality and the occurrence of postoperative complications (reintervention for bleeding, myocardial infarction, need for coronary revascularisation, stroke, perioperative dissection). Secondary endpoints during follow-up were the occurrence of type A and type B dissections, freedom from valve reintervention, freedom from valve or aortic intervention (ascending aorta and arch) and freedom from AR grade ≥2/4.

### Data analysis

Continuous variables were tested for normality with the Shapiro-Wilk test, shown as median (IQR) and compared using Mann-Whitney U test. Categorical variables were shown as n (%) and compared via Χ^2^ or Fisher’s exact test. Matched data were compared using the Wilcoxon signed-rank test or the McNemar’s (-Bowker) test for related samples. To adjust for potential confounders when comparing PEARS and VSRR, 1:1 PS matching was performed. We chose a PS analysis as it would yield two real populations after matching, rather than using a matching technique which would provide outcomes on a pseudo-population. Furthermore, we believed this strategy would help us better understand the overlap and discrepancies between the patients undergoing PEARS and VSRR. Gender, age, height, weight, a history of cardiac surgery, EuroSCORE II, left ventricular ejection fraction, maximal aortic root diameter, preoperative AR grade and scheduled concomitant procedure were used to determine individual PS, thereby including variables which we considered to be related to the underlying CTD severity. Underlying aneurysm aetiology was not used to match to avoid excluding patients with a less common CTD. There were no missing data among the covariates used in the PS model. Patients were matched using the ‘MatchIt’ package in R studio, performing a logistic regression to calculate the PS and matched with the nearest neighbour method, without replacement and a calliper width of 0.1 of the pooled SD of the logit PS. A calliper width of 0.1 was chosen as it resulted in optimal matching with standardised mean differences below 0.1 for all covariates as well as for the overall PS. Statistical significance was set at p<0.05. Survival, freedom from reintervention and freedom from AR ≥2/4 during follow-up were estimated using a Kaplan-Meier analysis with comparison between groups via a log-rank (Mantel-Cox) test. For patients undergoing PEARS, there was one echocardiography registration during follow-up and for VSRR patients there were between 1 and 26. Patients were censored after their last echocardiography and considered to have no occurrence of AR if there was no registration in the interval between registrations. Data analysis was performed using Microsoft Office Excel V.2016 (Microsoft), SPSS Statistics V26.0 (IBM) and RStudio (RStudio, PBC) and Prism (GraphPad Software).

### Patient and public involvement

No patients were involved in the design, conduct or reporting of this study.

## Results

### Baseline demographics

The 159 included PEARS patients were operated at 20 centres between 2004 and 2019, while the 142 patients undergoing VSRR from the AVIATOR underwent surgery at 13 centres between 1996 and 2021. Eighty patients in each cohort were matched using a PS analysis. Before matching, patients undergoing VSRR were significantly older, more likely to have mild AR or a history of cardiac surgery, had a higher EuroSCORE II and larger aortic root. After matching, covariates were balanced (table 1).

**Table 1:** Demographics for the total population and matched population

Demographics	Unmatched patients				Propensity score-matched patients			
	PEARS (n=159)	VSRR (n=142)	SMD	P value	PEARS (n=80)	VSRR (n=80)	SMD	P value
Age (years)	31 (22–40)	33 (26–41)	0.24	0.06	31.7 (21.5–42.5)	32 (26.25–38)	0.08	0.63
Male	101 (63.5%)	98 (69%)	0.12	0.32	57 (71.3%)	57 (71.3%)	0.00	1.00
Height (cm)	186 (180–193)	186 (180–194)	0.05	0.91	188 (182–194)	189 (182–194)	0.09	0.97
Aetiology				0.21				<0.01
Marfan	142 (89.3%)	117 (82.4%)			77 (96.3%)	64 (80.0%)		
Loeys-Dietz	15 (9.4%)	23 (16.2%)			2 (2.5%)	15 (18.8%)		
ACTA2 mutation	2 (1.3%)	2 (1.4%)			1 (1.3%)	1 (1.3%)		
EuroSCORE II (%)	1.0 (1.0–1.2)	1.6 (1.0–2.1)	0.43	<0.001	1.0 (1.0–1.2)	1.2 (1.0–1.6)	0.09	0.35
Previous cardiac surgery	3 (1.9%)	14 (9.9%)	0.27	<0.01	2 (2.5%)	3 (3.8%)	0.04	1.00
Root diameter (mm)	46 (43–48)	49 (46–50)	0.84	<0.001	48 (46–50)	48 (46–49)	−0.01	0.12
Preoperative AR grade			0.41	<0.001			−0.03	1.00
0/4	123 (77.4%)	81 (57%)			55 (68.8%)	56 (70.0%)		
1/4	36 (22.6%)	61 (43%)			25 (31.3%)	24 (30.0%)		
LVEF (%)			−0.06	0.65			0.00	1.00
Good >50%	148 (93.1%)	134 (94.4%)			76 (95.0%)	76 (95.0%)		
Moderate (31%–50%)	11 (6.9%)	8 (5.6%)			4 (5.0%)	4 (5.0%)		
Concomitant procedure planned	21 (13.2%)	26 (18.3%)	0.13	0.22	12 (15.0%)	13 (16.3%)	0.03	1.00

Underlying aetiology was not used to match. Continuous variables were compared using Mann-Whitney U test, categorical variables via Χ^2^ or Fisher’s exact test. Matched data were compared using the Wilcoxon signed-rank test or the McNemar’s test for related samples.

SMD was used to evaluate balance between groups.

AR, aortic regurgitation; LVEF, left ventricular ejection fraction; PEARS, personalised external aortic root support; SMD, standardised mean difference; VSRR, valve-sparing root replacement.

### Operative variables and in-hospital outcomes

In both groups, concomitant procedures predominantly involved the mitral valve (12.3%, 20 of 159 for PEARS and 11.3%, 16 of 142 for VSRR). Cardiopulmonary bypass was used in 18.3% (24 of 131) of uncomplicated isolated aortic PEARS cases. For patients undergoing VSRR, 57% were operated via the reimplantation/David technique and 37.4% underwent remodelling with aortic annuloplasty. Average aortic cross-clamp time was 138±33 min with nine patients requiring an additional clamping session. Nearly two-thirds (65.5%) of patients undergoing VSRR did not require cusp repair, while one cusp was repaired in 21.1%, most commonly via central free-margin plication. An overview of operative variables is shown in table 2.

**Table 2:** Operative variables

PEARS (n=159)	
PEARS completed	156 (98.1)
Isolated aortic PEARS	136 (85.5)
PEARS+mitral valve repair	18 (11.3)
PEARS+elective CABG	1 (0.6)
PEARS+mitral valve replacement	1 (0.6)
Converted to VSRR (1 with mitral valve repair)	2 (1.3)
Procedure aborted	1 (0.6)
Implant size (n=156)	
Scaled to 95% luminal diameter	77 (49.9)
Scaled to 100% luminal diameter	79 (50.6)
Completed PEARS procedures (n=156)	
Operative duration (min)	169 (145–204)
Isolated aortic PEARS (n=136)	
Operative duration (min)	164 (144–200)
CPB used	29 (21.3)
CPB time (min)	60 (41–77)

VSRR (n=142)	
Operative technique	
Reimplantation (David)	81 (57)
Remodelling+external annuloplasty	53 (37.4)
Remodelling (Yacoub)	8 (5.6)
Aortic leaflet procedures	
Tricuspid aortic valve	139 (97.9)
No repair	92 (64.8)
1 cusp repaired	28 (19.7)
2 cusps repaired	11 (7.8)
3 cusps repaired	8 (5.6)
Bicuspid aortic valve	3 (2.1)
No repair	1 (0.7)
1 cusp repaired	2 (1.4)
Undergoing concomitant procedure	26 (18.3)
Total concomitant procedures	29 (20.4)
Mitral valve repair	16 (11.3)
PFO closure	5 (3.5)
CABG	4 (2.8)
Ablation	2 (1.4)
Extra-anatomical coeliac trunk bypass	1 (0.7)
Tricuspid valve annuloplasty	1 (0.7)
Aortic cross-clamp time	133 (115–161)

CABG, coronary artery bypass graft; CPB, cardiopulmonary bypass; PEARS, personalised external aortic root support; PFO, patent foramen ovale; VSRR, valve-sparing root replacement.

In two patients scheduled for PEARS, an intraoperative conversion to VSRR was performed as the aorta was deemed too fragile (n=1), or after coronary injury occurred (n=1). As patients are entered into the AVIATOR Database by completed procedure, we did not capture conversions during intended VSRR. Seven (4.4%) patients undergoing PEARS needed an intraoperative or postoperative intervention for coronary injury or impingement (table 3). Three patients underwent coronary artery bypass graft (CABG) for injury to the right coronary artery (two without consequences, one suffered a myocardial infarction). One patient underwent intraoperative CABG for refractory ventricular fibrillation. In one patient, the longitudinal seam of the ExoVasc was urgently reopened due to right ventricular stunning. One patient underwent intraoperative CABG for left ventricular failure after mitral valve repair with concomitant PEARS. The left main stem was injured in one patient with a severe pectus deformity, as previously reported.^10^ This patient, in whom the PEARS procedure was aborted, died 6 days postoperatively from intracranial bleeding while on extracorporeal membrane oxygenation, resulting in a 0.6% early mortality for PEARS. Among patients undergoing VSRR, there were no early deaths, and one patient underwent postoperative stenting of the posterior descending artery. Ten (7%) patients in the VSRR group underwent a reintervention for bleeding or tamponade, while there were none after PEARS (p<0.001). An overview of in-hospital outcomes is shown in table 3.

**Table 3:** In-hospital and postoperative outcomes for total and matched population

	Unmatched patients			Propensity score-matched patients		
	PEARS (n=159)	VSRR (n=142)	P value	PEARS (n=80)	VSRR (n=80)	P value
**In-hospital outcomes**						
Reoperation for bleeding	0 (0%)	10 (7%)	<0.001	0 (0%)	6 (7.5%)	0.03
Coronary revascularisation	7 (4.4%)	1 (0.7%)	0.07	1 (1.3%)	0 (0%)	1.00
Stroke	1 (0.6%)	2 (1.4%)	0.60	1 (1.3%)	1 (1.3%)	1.00
Perioperative dissection	1 (0.6%)	1 (0.7%)	1.00	1 (1.3%)	0 (0%)	1.00
Perioperative death	1 (0.6%)	0 (0%)	1.00	0 (0%)	0 (0%)	–
Length of stay	6 (5–7)	7 (6–10)	<0.001	6 (5–7)	7 (6–9)	<0.001
AR grade postoperatively			<0.001			0.21
0/4	144 (92.3%)	100 (72.5%)		69 (86.3%)	62 (77.5%)	
1/4	12 (7.7%)	34 (24.6%)		9 (11.3%)	16 (20.0%)	
2/4	0 (0%)	4 (2.9%)		0 (0%)	0 (0%)	
**Postoperative outcomes**						
AV reintervention	1 (0.6%)	7 (4.9%)	0.12	0 (0%)	2 (2.5%)	0.28
AV/Asc/arch reintervention	3 (1.9%)	10 (7%)	0.26	1 (1.3%)	3 (3.8%)	0.67
Type A dissection	0 (0%)	0 (0%)	–	0 (0%)	0 (0%)	–
Type B dissection	1 (0.6%)	5 (3.5%)	0.26	1 (1.3%)	2 (2.5%)	0.5
Death	2 (1.2%)	4 (2.8%)	0.96	1 (1.3%)	3 (3.8%)	0.45
AR grade at last follow-up			<0.001			<0.01
0/4	129 (89.6%)	64 (49.6%)		64 (85.4%)	41 (57.8%)	
1/4	14 (9.7%)	51 (39.5%)		10 (13.3%)	25 (35.2%)	
2/4	1 (0.7%)	4 (3.1%)		1 (1.3%)	2 (2.8%)	
≥3/4	0 (0%)	10 (7.8%)		0 (0%)	3 (4.2%)	

Continuous variables compared using Mann-Whitney U test, categorical variables via χ^2^ or Fisher’s exact test. Matched data compared using Wilcoxon signed-rank test or McNemar’s test for related samples. When comparing Kaplan-Meier estimates, the log-rank test was used. Details on all reinterventions are shown in the online supplemental data.

AR, aortic regurgitation; Asc, ascending aorta; AV, aortic valve; PEARS, personalised external aortic root support; VSRR, valve-sparing root replacement.

10.1136/heartjnl-2022-321840.supp1 Supplementary data

### Survival

Median follow-up duration for PEARS patients was 25 months (IQR 12–52, total of 542 postoperative patient years), while for VSRR patients, it was 55 months (IQR 23–89, 713 postoperative years). No follow-up after discharge could be collected for two (1.3%) PEARS and four (2.8%) VSRR patients. Overall survival at 5 years was similar for PEARS and VSRR at 95.8% vs 99.2% (p=0.27). In the matched cohorts, survival was also similar at 5 years: 98.3% vs 98.6% (p=0.99) for PEARS and VSRR, respectively. Underlying causes of death and details on postoperative complications are shown in online supplemental table S1.

### Freedom from reintervention and aortic events

**Figure 2:**
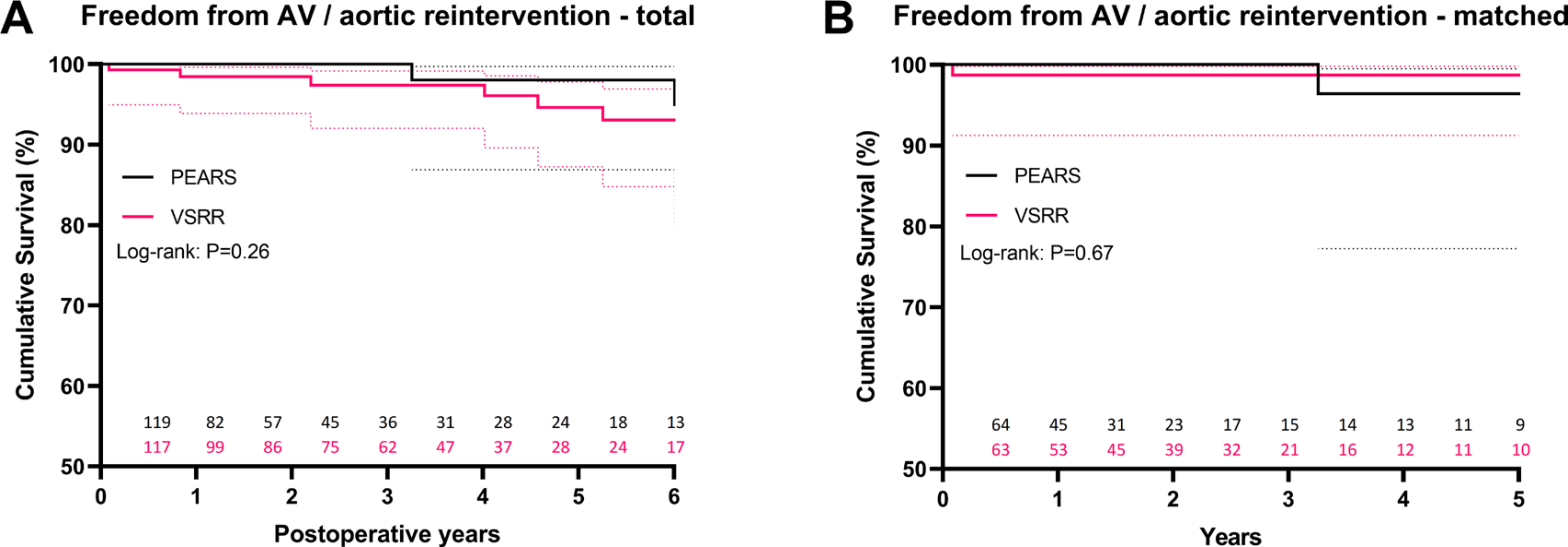
Kaplan-Meier estimate of freedom from aortic valve (AV) and ascending aorta/arch reintervention for total (A) and matched (B) cohorts, including 95% CIs. Graphs were truncated when less than 15% of patients remained at risk. PEARS, personalised external aortic root support; VSRR, valve-sparing root replacement.

Freedom from valve, ascending aorta and arch reintervention at 5 years was similar with 98% for PEARS vs 94.6% for VSRR (p=0.1) (figure 2). All three reinterventions in the PEARS group were related to operator failure to achieve complete coverage by the PEARS mesh, with one patient needing a total root replacement at 6 years after PEARS and two cases of off-pump redo-PEARS at 3 and 9 years. Reinterventions in the VSRR group were related to AR and/or progression of distal aneurysmal disease, or mitral valve pathology (online supplemental table S1). Among the seven patients who underwent an aortic valve reintervention after VSRR, six were initially operated via the remodelling technique, with or without an external aortic annuloplasty. In the matched cohorts, freedom from aortic valve or aortic reintervention was also similar at 5 years: 96.4% for PEARS vs 98.7% for VSRR (p=0.89). There were no type A dissections in either group, while there were five (3.5%) type B dissections after VSRR as opposed to one (0.6%) after PEARS (p=0.26).

### Aortic regurgitation

At last follow-up after PEARS (median 21 months, IQR 4–42), nearly 90% of patients had no AR and only one patient had an AR of 2/4. At last follow-up after VSRR (median 48 months, IQR 19–71), approximately 50% of patients had no AR while 10.9% had an AR of at least 2/4 (table 3).

Both preoperatively and at discharge, patients undergoing VSRR were significantly more likely to have mild (1/4) AR. After PS matching, there was no significant difference in AR grade at these time points (tables 1 and 3). Among matched PEARS patients, 85.4% had no AR and 14.6% had an AR grade of at least 1/4 at last follow-up (median 22 months, IQR 8–45). When comparing last echocardiographic follow-up of the matched PEARS group with intermediate follow-up after VSRR (median 22 months, IQR 5–35), patients who underwent VSRR had significantly higher AR grades: 61% had no AR and 39% had an AR grade of at least 1/4 (p=0.003).

**Figure 3:**
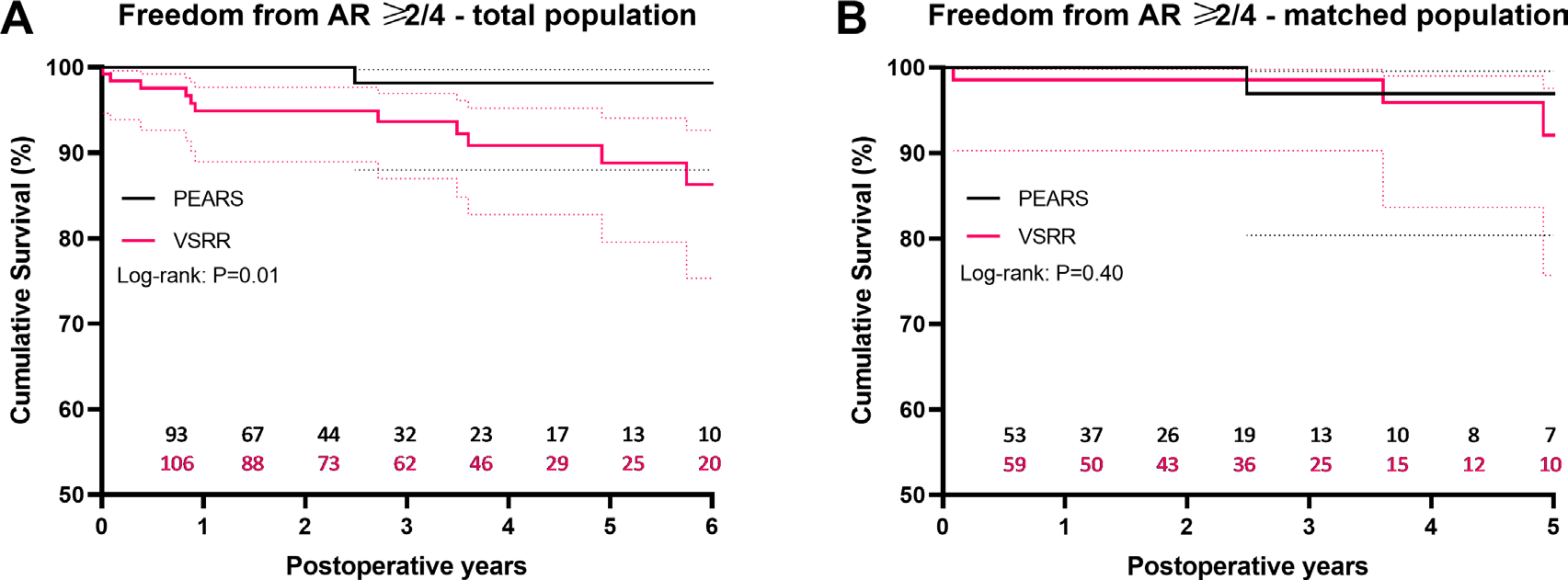
Kaplan-Meier estimate of freedom AR ≥2/4 for the total (A) and matched (B) population, including 95% CIs. Graphs were truncated when less than 15% of patients remained at risk. AR, aortic regurgitation; PEARS, personalised external aortic root support; VSRR, valve-sparing root replacement.

For the total population, freedom from AR ≥2/4 at 5 years was significantly greater after PEARS: 98.2% (n=23 at risk) vs 88.9% for VSRR (n=46 at risk) (p=0.02) (figure 3). In the matched population, there was no difference in freedom from AR ≥2/4 at 5 years (PEARS 97% vs VSRR 92.1%, p=0.55).

## Discussion

While the perioperative outcomes of PEARS and root replacement have been compared,^11^ this study represents the first side-by-side comparison of PEARS and VSRR evaluating safety, survival, freedom from reintervention and AR. Our observations fuel the relevant discussion on the challenges in referring and selecting patients with CTD for surgical treatment of their root aneurysm.^12^ In this subset of patients, both PEARS and VSRR aim to prevent ascending aortic dissection yet differ significantly in terms of surgical technique. PEARS is usually performed without use of cardiopulmonary bypass and preserves the blood–endothelial interface, while VSRR requires cardioplegic arrest and entails blood–prosthesis contact. PEARS is typically used in patients with at most mild AR and at a smaller root diameter than at which root replacement would be indicated.^7^

Our study cohort was derived from real-world clinical data and consists of a selected population of young patients with predominantly MFS, normal ejection fraction and at most mild AR undergoing elective surgery. The PEARS 200 Database represents the most extensive follow-up on PEARS to date, starting from patient 1 as the procedure was disseminated worldwide.^7^ The AVIATOR Initiative is an open international registry, including over 5000 patients by now, aiming to define the role of aortic valve repair in patients with AR and ascending aortic aneurysm.^9^ Our study needs to be interpreted in light of the challenges associated with comparing two retrospective databases with different duration of follow-up.

Patients undergoing VSRR were further along in their disease progression or had a more severe CTD phenotype than those undergoing PEARS. Most remarkable were differences in age, root diameter, EuroSCORE II and preoperative AR (table 1). Patients for whom the surgeon judged there was an indication for aortic root surgery at diameters below conventional thresholds may have additional risk factors, yet we did not have data on clinical decision-making available.^1^ The median aortic root diameter of 49 mm in our selected population of patients with MFS undergoing VSRR was similar to the diameter of 48–54 mm reported in earlier series, depending on selection criteria per centre.^13 14^ Among patients undergoing VSRR, 57% had no AR preoperatively and repair of at least one cusp was performed in 34.5%, most commonly via a central plicating stitch. Renowned centres report a highly selective approach to VSRR in MFS with 83%–85.6% of patients having at most mild AR and cusp repair performed in 10%–20%.^13 15^

With no perioperative deaths and a median EuroSCORE II of 1.6, VSRR was safe in this study, yet 7% of patients required a reintervention for bleeding. There was a 4.4% incidence of coronary complications observed with PEARS, including one death. This may be partially related to the learning curve of this emerging procedure, reflected by the fact that these events occurred early in the experience. On the other hand, this issue may not be wholly abolished as the procedure is technically challenging.

In this selected group of patients, both PEARS and VSRR seem to offer favourable and statistically similar mid-term survival and freedom from reintervention, both in the total and matched populations. While the reintervention rate after PEARS was 0.55% per year, the reintervention rate for VSRR was 1.4% per year, higher than the 0.6% per year found in a recent meta-analysis on the outcomes of root replacement in MFS.^4^ There was no difference in freedom from AR ≥2/4 at 5 years between matched groups, yet patients undergoing VSRR had significantly higher AR grades at last follow-up (table 3). While this could be related to longer follow-up in the VSRR group, the risk of developing AR is inherent to VSRR.^16^ In PEARS, aortic root geometry is preserved or slightly downscaled by a 95% luminal-diameter-scaled implant, likely improving leaflet coaptation.^17^ VSRR, on the other hand, entails a significant reduction of aortic root dimensions, thereby acutely altering leaflet configuration, potentially leading to cusp prolapse.^16^

The absence of type A dissections after PEARS in 159 patients with 542 years of follow-up indicates that this procedure has the potential to prevent aortic dissection, yet continued follow-up is needed. The elevated risk of type B dissection and reintervention, while not significant, in patients undergoing VSRR suggests that earlier intervention or more aggressive management of the distal aorta may be indicated. Furthermore, a stiff interposition graft as used in VSRR causes a marked increase in wall stress distally, while the PEARS mesh becomes incorporated histologically and has a gradual reduction in hoop strength from proximal to distal.^18–21^

There are several important limitations to our study. This retrospective, multicentre study used two different databases, including 301 patients operated at 33 centres between 1996 and 2021. While outcomes after PEARS were reported according to intention to treat, data were extracted from the AVIATOR Database per completed procedure. We could therefore not identify patients who underwent an intraoperative conversion during VSRR. Due to low event rates and differences in follow-up, our description of late outcomes was predominantly descriptive. While the outcomes of VSRR are influenced by procedure type (reimplantation and remodelling with or without external annuloplasty in this study) and patient selection, our study does not factor procedure type into the comparison with PEARS. As we did not have data on aortic valve morphology in the PEARS group, we were unable to compare patients accordingly. Furthermore, we only had data on one late echocardiography for PEARS patients while VSRR patients had more extensive follow-up. Our echocardiographic data should be interpreted in the setting of a multicentre study. Using a PS analysis, we aimed to quantify differences between patient populations and correct for selection bias between groups, yet, are unable to determine whether differences in AR grade after PEARS or VSRR are related to uncorrected confounders, different follow-up duration or the development of AR after VSRR. Using our matching approach, patients with outlying aortic root diameters may be excluded. We focused on variables indicative of disease severity while not including CTD phenotype in the PS model as we did not have data available on the underlying genetic mutations which are strongly related to disease severity.^22^

### Conclusions

VSRR and PEARS both seem to offer favourable mid-term survival, freedom from reintervention and preservation of valve function in syndromic root aneurysm. Depending on the disease stage of the individual patient, both treatments may be complementary, yet long-term follow-up is needed for PEARS. Our study indicates that an earlier intervention in the disease progression via PEARS may be justified with a low probability of developing aortic events if the necessary attention goes to the coronary anatomy. Improved prediction of aortic complications is needed to help us guide patient selection. A shared decision-making process should involve weighing the risk of watchful waiting against the potential risks of all available surgical treatments.

10.1136/heartjnl-2022-321840.supp2 Supplementary data

## Data Availability

Data are available upon reasonable request.
