# Supplementary material for: Matched comparison between external aortic root support and valve-sparing root replacement
Source: Heart. 2023 Jan 17;109(11):832–8. doi: 10.1136/heartjnl-2022-321840 (PMC10313978; doi:10.1136/heartjnl-2022-321840)
Supplement: Supplementary data [file heartjnl-2022-321840supp001.pdf]

Supplementary data

Table S1. Detailed perioperative and postoperative outcomes. Symbols ¹ and ² refer to the same 2 patients. *: underwent reintervention on both aortic valve and the aorta. °: this patient first underwent a reoperation on the aortic valve, and later on the mitral valve. Continuous variables compared using Student's t-test, categorical variables compared using Chi square, Fisher's exact test or Log Rank test as appropriate. Ao Asc=ascending aorta, AS=aortic stenosis, AR=aortic regurgitation, FET=frozen elephant trunk, MVP=mitral valve plasty, RCA=right coronary artery, TEVAR=thoracic endovascular aneurysm repair, TRR=total root replacement.

IN-HOSPITAL OUTCOMES	PEARS (n=159)	VSRR (n=142)	P-value
**Intraoperative conversion**	2 (1.2%)<br>1 VSRR - very thin aorta<br>1 Florida sleeve - haematoma on RCA	0 (0%)	1.00
**Reoperation for bleeding**	0 (0%)	10 (7%)	<0.001
**Coronary revascularization**	7 (4.4%)<br>3 CABG after RCA injury<br>1 mesh reopened urgently for RV stunning ¹<br>1 CABG, ECMO after coronary injury ²<br>1 CABG due to VFib<br>1 CABG for LV failure after PEARS + MVP	1 (0.7%)<br>1 stent to right coronary artery	0.07
**Myocardial infarction**	3 (1.9%)	0 (0%)	0.25
**Stroke**	1 (0.6%)	2 (1.4%)	0.60
**Perioperative dissection**	1 (0.6%)	1 (0.7%)	1.00
**Perioperative death**	1 (0.6%) ²	0 (0%)	1.00
**AR grade postop**			<0.001
0/4	144 (92.3%)	100 (72.5%)	
1/4	12 (7.7%)	34 (24.6%)	
2/4	0 (0%)	4 (2.9%)	
**Length of stay (d)**	6 (5 - 7)	7 (6 - 10)	<0.001
**POSTOPERATIVE OUTCOMES**	**PEARS (n=159)**	**VSRR (n=142)**	**P-value**
**Cardiac / aortic reintervention**	3 (1.9%)	11 (7.7%)	0.17
**Aortic valve reintervention**	1 (0.6%)<br>1 TRR because mesh was reopened urgently (6y) ¹	7 (4.9%)<br>1 endocarditis (1m)<br>4 due to AR (1, °2, 4 and *16y)<br>1 due to mixed AS/AR (8y)<br>1 TRR and Ao Asc replacement (14y) *	0.12
**Cardiac / Ao Asc / Arch reintervention**	3 (1.9%)<br>1 redo-PEARS for faulty use - incomplete coverage (3y)<br>1 TRR because mesh was reopened urgently (6y) ¹<br>1 redo-PEARS for bulge around RCA (9y)	7 (4.9%)<br>1 for mitral valve endocarditis (4m)<br>1 for mitral para-prosthetic leak (3y) °<br>2 Ao Asc & Arch replacement + FET (4 and 5y)<br>1 debranching + TEVAR (6y)<br>1 TRR and Ao Asc replacement (14y) *<br>1 TRR + Ao Asc + Arch + debranching (16y) *	0.71
**Type A dissection**	0 (0%)	0 (0%)	-
**Type B dissection**	1 (0.6%)	5 (3.5%)	0.26
**Death**	2 (1.2%)<br>1 unknown (1y)<br>1 arrhythmia (4y)	4 (2.8%)<br>2 non cardiac (2m and 18y)<br>1 unknown (13y)<br>1 post-aortic reintervention (5y)	0.96
**AR grade at last follow-up**			<0.001
0/4	129 (89.6%)	64 (49.6%)	
1/4	14 (9.7%)	51 (39.5%)	
2/4	1 (0.7%)	4 (3.1%)	
3 and 4/4		10 (7.8%)	
