# Supplementary figures and images for: Matched comparison between external aortic root support and valve-sparing root replacement
Source: Heart. 2023 Jan 17;109(11):832–8. doi: 10.1136/heartjnl-2022-321840 (PMC10313978; doi:10.1136/heartjnl-2022-321840)

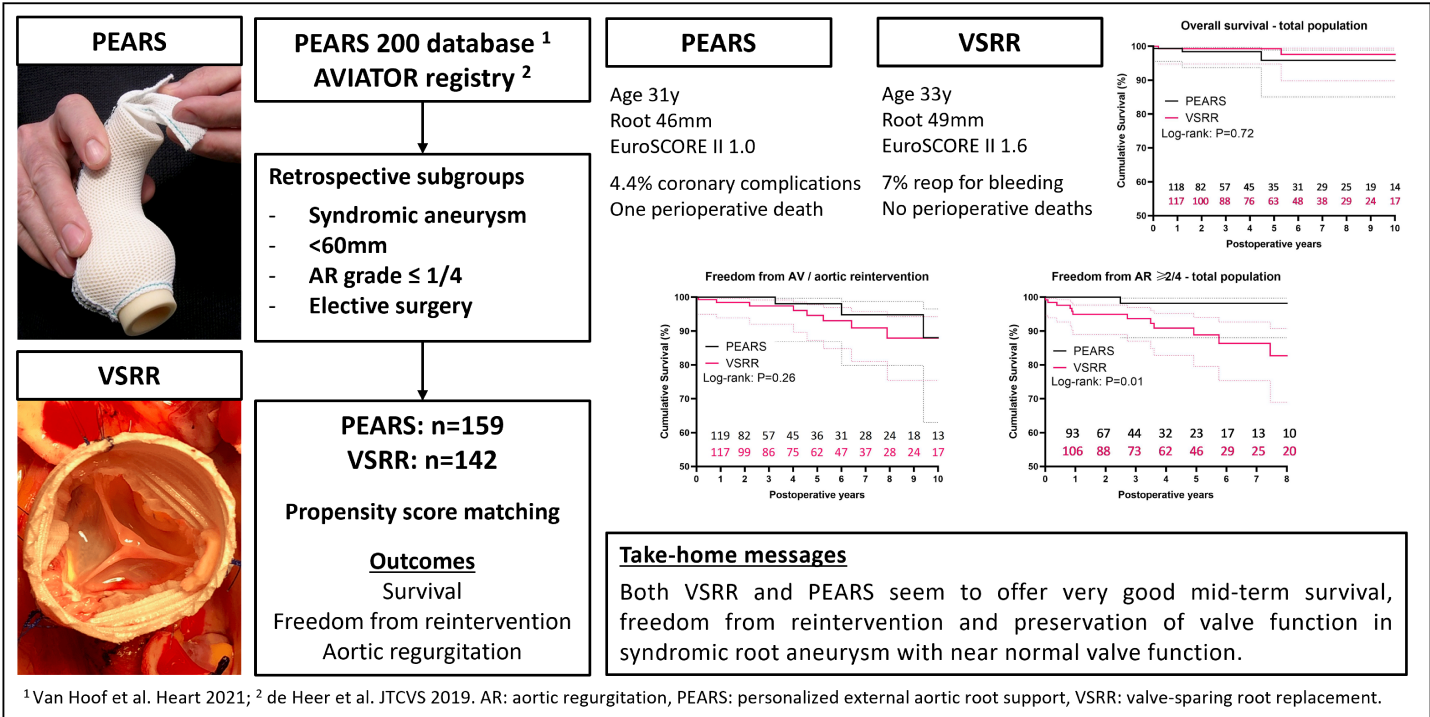

Supplement: Supplementary data [file heartjnl-2022-321840supp002.pdf]
